# Statistics of Insanity

**Published:** 1861-09

**Authors:** 


### Bi-Monthly Periscope.

### STATISTICS OF INSANITY.

### 1. FRANCE.

M. LEGOYT, who is at the head of the French Bureau of General Statistics, published, a few months ago, a volume of statistics relative to establishments for the insane in France. The following analysis of this work, from the pen of M. Brierre de Boismont, presents a comprehensive summary of the lunacy statistics of France, and is extracted from the last number of the *Annales d'Hygiène Publique.* We have omitted that portion of M. Boismont's analysis which refers to expenditure and deficiencies in asylum accommodation:—

Notwithstanding the efforts made by the French administration to obtain the most complete information respecting the insane under treatment, exact data are still wanting. Idiots and cretins were not distinguished in the returns until the year 1853; and there is an excess of at least 300 per annum in the figures indicative of the total number of insane, in consequence of duplicate returns. Both these sources of error will, in future, be removed.

On the 31st December, 1853, the number of lunatic establishments amounted to 111. Of these 65 were public and 46 private. One of the public asylums belongs to the State, (Charenton,) 37 to the departments, 1 to the communes, and 26 are ancient religious houses or foundations, or divisions of a civil hospital, (*hospices ou quartiers d'hospices.*) Among these 111 establishments, 11 are devoted specially to males, 17 to females, and 83 receive both sexes. Twenty-five departments have still no establishments specially set apart for the insane.

In 1835 the number of insane under treatment was ascertained for the first time. Since this period, the figures have increased year by year with one exception, in 1850, during which year a decrease occurred in consequence of the mortality from cholera in 1849. The number of insane in the different asylums on the 1st of January in each year from 1835 to 1854, inclusive, was as follows:—

| | | | | | |
|---|---|---|---|---|---|
| 1835 . . | 10·539 | 1842 . . | 15·280 | 1849 . . | 20·231 |
| 1836 . . | 11·091 | 1843 . . | 15·796 | 1850 . . | 20·061 |
| 1837 . . | 11·429 | 1844 . . | 16·255 | 1851 . . | 21·253 |
| 1838 . . | 11·892 | 1845 . . | 17·089 | 1852 . . | 22·495 |
| 1839 . . | 12·577 | 1846 . . | 18·013 | 1853 . . | 23·795 |
| 1840 . . | 13·283 | 1847 . . | 19·023 | 1854 . . | 24·524 |
| 1841 . . | 13.887 | 1848 . . | 19·570 | | |

According to this table, it would appear that the population of asylums, from the 1st January, 1835, to the 1st January, 1854, has more than doubled. It has increased in nineteen years 13,985, that is, about 1·33 per cent.

From 1842 to 1854 the number of male lunatics was 9314; of female, 10,177. At the first glance we might be tempted to conclude from this difference that women had a peculiar predisposition to insanity; but as the females under treatment in asylums are more numerous than the men, the excess is dependent upon the sojourn of the latter being less than that of the former, and upon the mortality being greater among males than females.

The 23,795 insane existing in the asylums on the 1st January, 1853, were thus distributed: in the asylums of the State, the departments, and the communes, 10,839; in the *quartiers des hospices*, 7233; in private asylums, 5733. This gives for the first, 45·55 per cent.; for the second, 30·36; and for the last, 24·09.

On investigating the relation of the number of insane under treatment to the total population of France, at five quinquennial periods, the following results are obtained:—

| | | | | | | |
|---|---|---|---|---|---|---|
| 1836 . . | 33,540,910 population, | 11,091 insane; | 1 | in every | 3,024 | individuals. |
| 1841 . . | 34,240,178 " | 13,887 " | 1 | " | 2,465 | " |
| 1846 . . | 35,400,486 " | 18,013 " | 1 | " | 1,965 | " |
| 1851 . . | 36,783,170 " | 21,353 " | 1 | " | 1,676 | " |

From these figures we ascertain that, in relation to population, the number of insane under treatment has augmented considerably; for while the increase of the population from 1836 to 1851 has been 6·68 per cent., the increase of the insane has been 92·52 per cent., or nearly fourteen times as much.

But the number of insane under treatment in asylums is not an exact representation of the real number which exists; there are, indeed, many more of these unfortunates than are to be found under treatment. During the enumeration of the population in 1851, it was ascertained that 24,433 individuals deprived of reason were living in private dwelling-houses; and if this number be added to the population of the asylums in that year, we obtain a total of 44,970 insane, that is, 1·25 in every 1000 inhabitants, or 1 in 796. When the last census was taken in England, it was found that one-half of the demented individuals in that country were deprived also of the aid that they required, and contributed to augment the contingent of mental alienation. It is to be remarked that the departments (25 in number) which contain the greatest amount of insane in private dwellings are those in which no asylums exist.

From 1842 to 1853, with the exception of 1849, (the cholera year,) there was an excess of admissions into the asylums, as compared with the united discharges and deaths, the excess amounting to an annual mean of 766. If this proportion of admissions continue, it will be requisite every year to provide new asylums capable of accommodating the mean excess named, or to enlarge to the requisite extent the asylums already existing. The actual number of individuals received into establishments devoted to the insane, during the period named, was 94,169; 52,871 were dismissed, and 32,099 died. These figures give the annual mean of 7847 admissions to 4406 discharges and 2675 deaths; or 11 admissions to 10 discharges and deaths.

Of 1000 admissions, 533 were males, and 467 females; of 1000 discharges, before or after cure, 535 were males, and 465 females; and of 1000 deaths, 541 were males, and 459 females: consequently, the admissions being equal, there die in lunatic asylums many more men then women; and, on the other hand, the sojourn of the latter is notably longer than that of the men. This is, as has already been said, the explanation of the numerical predominance of the female sex in the actual population of asylums.

The mean annual number of admissions, discharges, and deaths, in each of the different classes of asylums from 1844–52 inclusive, was as follows: (1) In asylums appertaining to the State, the departments, or the communes, 5717, (admissions, 3138; discharges, 1511; deaths, 1068.) (2) In hospital establishments, 6049, (admissions, 3066; discharges, 1937; deaths, 1046.) (3) In *maisons de santé*, 3274, (admissions, 1752; discharges, 956; deaths, 566.)

The continuous increase witnessed in the number of insane under treatment is equally remarked in the number of annual admissions: thus the figure which in 1835 was 3947, arose in 1853 to 9081. During this period of nineteen years the total number of admissions was 128,542. In 1852 the total number of admissions was greatest; if it be compared with 1835, it will be found that the admissions have nearly trebled in eighteen years.

Without prejudging the question of the influence upon this increase of the number of asylums, of their enlargement, of the ameliorations which they have undergone, or of the action of civilization upon the development of insanity, M. Legoyt presents some facts which appear to him not without interest in the solution of the problem.

He observes that, if we divide the nineteen years comprised between 1835 and 1853 into four periods of from three to five years each, we shall find that the mean number of annual admissions has been as follows:—

| | | | |
|---|---|---|---|
| For the 1st period | (1835–1838) | . . . . . . | 4378 |
| " 2d " | (1839–1843) | . . . . . . | 6061 |
| " 3d " | (1844–1848) | . . . . . . | 7510 |
| " 4th " | (1849–1853) | . . . . . . | 8635 |

The mean increase from period to period, as deduced from these figures, is as follows:—

| | | | | | |
|---|---|---|---|---|---|
| From the 1st to the 2d period | 38·44 per cent., | or | 9·61 | per cent. | per annum. |
| " 2d " 3d " | 23·91 | " | 4·78 | " | " |
| " 3d " 4th " | 14·97 | " | 2·99 | " | " |

From this table it would appear that since 1835 the increase of admissions gradually diminishes, in such a manner that the time may be foreseen when, all things being equal, the annual number of admissions will become stationary. It may also be remarked that if civilization, in raising the level of general comfort, has a tendency in divers ways to excite suffering, it neutralizes by degrees the deadly consequences of misery upon the public health. The increasing number of admissions can be explained by considerations every way foreign from physiological reasons: the creation of new asylums, the amelioration of the internal *régime* of these establishments, the extension of moral treatment, the reputation of physicians, the diminution of the prejudices respecting insanity, the moderate charges of the establishments, the rapidity of communications, and the gratuitous charge of indigent lunatics! It must also be mentioned, that lately abuses have affected the admissions, in consequence of the municipal authorities and even families imposing upon the departments, under the pretext of mental derangement, the charge of a great number of paupers. We shall not discuss these points, but confine ourselves to the remark that everywhere where the human brain is without cessation kept in action, there we are sure to see the number of insane predominate: it is thus that in France, in England, in the United States, where the physiological causes are excessively multiplied, the number of insane is considerable, while in Italy and in Spain the proportion is much less: it is also small in Turkey and in Asia. As to the predominance of moral causes above physical, we consider it incontestable; but it is necessary to know that the cause may be hid with infinite care, and that it is not in mere passing relations we shall obtain such avowals as these: "I have failed in my duties to my spouse;" "I have committed perjury;" "I have seduced the sister of an intimate friend, and he has perished before my eyes," etc., etc. Even M. Legoyt admits that there are thousands of fools who are not treated as such, and I would add that many are carefully hidden. Lastly, the alliances between relatives, and between insane individuals, tend without ceasing to propagate the malady. M. Legoyt says that it has been demonstrated that, during the great social crisis of 1848, there was a diminution in the number of admissions. We answer that many of these unhappy individuals fell victims to their exaltation; others fled the country; and the prisons received a large number. It has been a matter of observation, during many years, that a notable quantity of victims to political crises have entered into lunatic establishments. Moreover, it would be necessary to count those who, conceived under the influence of great disturbances, subsequently became insane.

On comparing the annual admissions with the population, we wrote that, during this period of 12 years, there has been in the department of the Seine 1 admission in every 516 inhabitants, while the proportion for the whole of France has been 1 in 4144. This result is explained by the exceptional state of the City of Paris, which obliges the authorities to sequestrate every individual deprived of reason; by the just celebrity of many establishments for the insane; and by the facilities afforded to families for having their insane treated there in absolute secrecy.

The relation of the sexes ought to be examined with care. It would appear from M. Legoyt's documents, that the admissions of men have exceeded those of women in a proportion which averages more than 14 per cent. Now, it is necessary not to lose sight of the fact that there are more women than men in the total population; hence there is a certain degree of probability that man is more predisposed to insanity than woman. According to the enumeration of 1851, and the annual mean of admissions during the quinquennial period 1849–53, it would appear that, for the whole of France, there were 3864 individuals of the male sex to every male admission, and 4473 of the female sex for every female admission.

The admission into an asylum may be carried into effect either by the families of lunatics, or by the administration, which intervenes officially. Of the 9081 patients cured in all the establishments in 1853, 2609 were placed under charge by their friends, and 6472 by the administration: thus more than two-thirds of the admissions were exacted for the public security. In the Parisian asylums, the proportion of cases sent by the administration is nearly 80 per cent.

In respect of age, from the fiftieth to the sixtieth year females are attacked more frequently than males; in 1000 cases of each sex, the number of instances within the period named being 134 males and 167 females. This result appears to indicate the influence of the critical time. As to the mean age of admission, it is 40 years and 5 months. One of the most remarkable facts made manifest by the returns respecting the civil condition, is the large number of unmarried people among lunatics in asylums; the percentage, of both sexes combined, upon the total number of insane under charge, being 61·80; while in the population at large, above the age of fifteen, the unmarried form only 36·74 per cent. This apparent predisposition to insanity among the unmarried has been attributed to the absence of those joys and cares which belong to a family, and to the terrible trials of adversity suffered alone. Without denying these reasons, M. Legoyt, however, thinks that it is necessary also to take into consideration the isolated state of the unmarried lunatic, which renders it necessary that he should be promptly conducted to an asylum.

In regard to professions, M. Legoyt has shown that, in proportion to their numbers, artists, in 1853, numbered eight times more insane than proprietors and landlords; Jesuits, seven times more; ecclesiastics and physicians, five times more; professions and men of letters, four times. For these five categories of the population combined, there were 205 individuals for one lunatic under treatment; while for the entire population, 1294 inhabitants are found for one lunatic. Soldiers and sailors must be put aside altogether, because, being sent without exception, by authority, to a special establishment, there cannot be a comparison established between them and other classes of the population, of which a great number are never placed in asylums. This result confirms the opinions generally entertained, that the professions which exact continual brain-work contain a greater number of insane than others. After trades come domestics or journeymen, the manual or mechanical professions. The proportion of insane belonging to the category of domestics and of journeymen exceeds the half of the general mean: it is also in this class that the greatest number of unmarried persons is found.

The degree of instruction among the insane has been the object of examination; but as there are no data respecting the amount of instruction existing among the entire population, the results have no relative value. Considering, however, the statistics in a general point of view, it is evident that that portion of the population of which the instruction is superior to that which has rudimentary instruction merely, furnishes a considerable contingent to the number of insane treated, since it forms nearly a twelfth. This proportion is nearly equal to that of the liberal professions.

Of 2883 lunatics, in 1853, hereditary predisposition is stated to have existed in 1410 men and 1470 women. Of 1000 lunatics, the cause of insanity was said to be physical in 572, and moral in 428. We have already remarked upon the necessity of living in intimacy with the insane in order to obtain a correct knowledge on this subject, and that the inexact information obtained in asylums, both public and private, reduces very much the value of the figures referring to heritage and other causes. There are also other objections in regard to the physical causes, because it is evident that drunkenness, bereavement, and misery have a double meaning. The man who, for example, drinks to stupefy his grief, and becomes insane, at first acts under the influence of a moral cause. Accidental suppression of menses (150) and puerperal insanity (150) in a great number of cases arise from moral impressions.

Among moral causes, the most frequent is grief arising from the loss of money: 899 cases of insanity are attributed to this cause, which is, by comparison with the total figure of moral causes, a proportion of more than 12 per cent. Afterward come religious exaltation, 1894; love, 792; violent emotions, 698; pride, 600; the loss of a loved one, 510; disappointed ambition, 495; jealousy, 442; political events, 308; excess of intellectual work, 156; *simple* imprisonment, 154; nostalgia, 48; isolation and solitude, 41; change of life, 32; association with and assiduous attention upon the insane, 16; *cellular* imprisonment, 4.

When the admissions are arranged according to seasons, it is seen that the months of summer and of autumn are those which are most charged. For both sexes, and for the female sex, the maximum of admissions takes place in July; for the male sex, in November. This difference is indicated now for the first time.

An interesting point for study is the duration of insanity at the time of reception into an asylum. Of 14,963 insane respecting whom information was furnished, in 1853, it is noted that nearly half the number had been suffering more than two years. If this return be exact—and we believe, from our own experience, that it is so—we need not be surprised at the number of insane who cumber our asylums. The English returns direct attention every year to this sad result—the result of ignorance, cupidity, and indifference.

Idiots and cretins were distinguished for the first time in the statistical returns for 1853. In that year the number of idiots is stated to have been 2654, of cretins only 45. We may remark concerning this last figure that it must be very far from being correct, for it is not in relation with the great number of existing cretins, of whom it has been estimated 3000 exist in the department of the Basses-Alpes alone.

Insanity has been considered one of the maladies which offers the greatest number of relapses. Of the 32,876 insane which form the object of this examination, the relapses amount to 1635, being a proportion of 49 in every 1000 insane. It will be acknowledged that this proportion is much less than that which occurs in many other affections, and particularly in rheumatism.

Of the 32,876 insane returns in 1853, comprehending the 23,795 in the asylums on the 1st of January in that year, and the 9081 received during the year, 12,972 came from towns, 14,536 from the country, and of 5368 the residence was unknown. Now, as the population of the towns is to that of the country as 1 to 3, it follows that the urban insane are much more numerous than the rural. This result has been attributed to the development of luxury, extreme covetousness, agitations, excesses, disorders of all kinds, industrial crises, and the miseries that they lead to. M. Legoyt, however, would attribute it to differences in administrative measures. Thus, while in towns the insane are most commonly placed in confinement as dangerous, in the country the inoffensive are left to the charge of their families; from which it follows that the towns ought to show a marked numerical superiority of insane under treatment as compared with the country. This opinion is supported by the results of the census of 1851, which show 1856 insane in 3632 cities and chief towns of *arrondissements*, and 22,577 in the communes. But it is to be observed that researches made with care by the directors of asylums demonstrate the predominance of the number of insane in towns in relation to the amount of population. There is, also, another fact which belongs to this question, that is, the excess in number of suicides occurring in towns as compared with the country, and we know the intimate relation which exists between suicide and folly.

It is difficult to appreciate rightly the results afforded by the returns of cures, because the figures vary singularly according as establishments receive patients curable or incurable, as at Bethlehem or Hanwell. In France the asylums admit indiscriminately all cases, and the proportion of chronic cases is enormous. Some asylums make their returns solely upon curable cases, while others take the total number of admissions. To obtain an exact number of cures, it would be necessary to put aside paralytics, epileptics, demented, chronic cases, idiots, cretins, *caput mortuum* of which the lot is fixed in advance, and to hold count only of recent cases of mania and monomania, the sole which have a chance. In 1853, of 4872 cases discharged, 2771 were cured and 2101 not cured. Relatively to the duration of treatment in the cases cured, 36 per cent., or more than one-third, were cured within the first three months of treatment; 25 per cent., or one-fourth, after six months' treatment; 11 per cent., or about one-tenth, in from six to nine months; 8 per cent. in from nine to twelve months. This gives, consequently, 80 per cent. of cures within the first year, and 20 per cent. only in subsequent years. The mean duration of the cures was nine months fifteen days; and the mean age of the individuals cured, thirty-seven years two months, for both sexes.

During the period of twelve years comprised between 1842 and 1853, there died 32,099 individuals in lunatic asylums, or an annual average of 2675. Of this proportion, 17,390 were men, and 14,709 women.

If the mortality be compared in public and private asylums, we obtain the following results:*—

| | | | | |
|---|---|---|---|---|
| Departmental asylums . . . | 1 | death in 7·90, | or 12·66 | per cent. |
| In hospital establishments . . | 1 | " | 6·45, " 15·60 | " |
| In *maisons de santé* . . . . | 1 | " | 8·10, " 12·35 | " |

Under the denomination *maisons de santé* are included establishments which receive as many as 100 insane, of whom the greatest part might also be placed in the *hospices:* be this as it may, the mortality in the *quartiers d'hospices* is the most considerable. This is to be attributed to these establishments being old constructions, badly situated in the middle of towns, and not fitted for the purpose to which they are devoted. In 1853 sixteen accidental deaths and seventeen suicides occurred. This last figure is not surprising, if we reflect on

* It is not stated how these results were obtained.—ED.

the frequency of suicidal mania. We may add even that it is providential, when we know how fixed the resolutions of the suicidal insane are. Esquirol tells us that when a lunatic wishes to kill himself, he does it in spite of precautions: this is also our conviction.

All those who have the direction of public or private asylums know the large proportion of deaths during the first month of admission. According to M. Legoyt, it reaches 10·8 per cent., or more than a tenth of the whole mortality; and he asks if, independently of the states of debility stated by some alienists as to the cause of mortality, the sudden change of regime, and the violent emotion occasioned by this hasty sequestration, may not have an unfavorable influence? We are astonished that emotion should be assigned as a cause, because for thirty years that we have been constantly in contact with the insane, and that we have observed them with particular care, we have never seen emotion occasion a grave accident. The immense majority of the insane have not a knowledge of their state; they are generally egoists; many, without doubt, regret their liberty, beg for it, and make attempts to escape; but they are rarely attacked with nostalgia, and when this does occur, the dismissal takes place nearly always immediately. The mortality of the first month, then, must be attributed to other causes, and these are those which we have noted and which our *confrères* have noted also. A great number of cases kept a long time at home are placed in asylums only when they become noisy or refuse every attention: this happens often with general paralytics; and this state corresponds always to a period of aggravation or of fatal termination. Acute and grave cases, such as acute delirium, with obstinate refusal to take food, from fear of being poisoned by enemies, terminates also unfortunately in a few days, when the aid of art is insufficient. Many insane, treated at home, from some motive or other are sent into an asylum to die there. Lastly, it frequently happens that sick are sent to asylums who are suffering from the delirium of typhoid and ataxic fevers, encephalitis, pneumonia, etc., and who expire a few hours after admission, or at the end of two or three days. This rapid enumeration, which does not comprehend every case, gives a satisfactory scientific explanation of the elevated figure of the mortality in the first month.

The seasons have upon the mortality of the insane the same influence that they have upon the mortality of the total population of France.

### 2. IRELAND.

The Appendix to the Report of the Commissioners of Inquiry into the State of the Lunatic Asylums, and other institutions for the custody and treatment of the insane in Ireland, contains a series of statistical tables relative to the district asylums, from which several interesting particulars may be obtained respecting the recoveries, mortality, and character of the cases received into those institutions. In our last number we gave an account of the nature and tendency of the Commissioners' Report; we now propose to lay before our readers a few of the principal results which may be gathered from the Appendix.

In our previous notice of the Report, we gave a summary of the general statistics contained in it, but we may quote here the following statement of the

Commissioners: "The Census Commissioners (1851) and the Inspectors of Lunatics in their last Report have given returns of the total number of insane in Ireland in 1851 and 1857 respectively. The returns of the inspectors are not given for the same date in the several institutions, and they contain, moreover, the number of persons simply 'epileptics' at large and in the workhouses; and as these are exclusive of the 'lunatics' and 'idiots' in those returns, they may be omitted from the total of the insane. The Census Returns give the number of insane in Ireland on the 31st March, 1851, at 9980, of whom 4635 were at large. The Inspectors of Lunatics, in their last Report, (if we exclude epileptics at large and in workhouses, for those in asylums must be supposed to be insane,) fix the number at 11,452, of whom 5441 are at large. A comparison of these returns, made at an interval of between five and six years, and obtained through the same sources of information, shows a considerable increase in the amount of insanity in Ireland."—*Report*, p. 2.

During the five years 1852–56, 6197 cases (3249 males and 2948 females) were admitted into the district asylums, and the daily average of patients during the period was 3467. The total *discharges* amounted to 3715, (1936 males, 1779 females,) the *recoveries* being 2535, (1237 males, 1298 females,) and the *non-recoveries* 1280, (699 males, 581 females.) The total *mortality* was 1363, (722 males and 641 females.) If the proportion of recoveries and deaths be calculated upon the admissions, it is found that the average ratio of the former during the five years was 39·2 per cent., of the latter 10·5 per cent.; but if the more correct calculation suggested by Dr. Farr be made, the proportions being calculated upon the mean of the admissions, discharges, and deaths—this mean giving the nearest approximation to the number of cases treated—the following results, *per cent.*, are obtained: *Recoveries*, males, 21·9; females, 21·2—total, 43·1. *Deaths*, males, 12·8; females, 10·3—total, 22·7.

The distribution of the deaths in the different months was as follows:—

| | | | |
|---|---|---|---|
| January | 125 | July | 57 |
| February | 106 | August | 107 |
| March | 147 | September | 76 |
| April | 125 | October | 124 |
| May | 127 | November | 101 |
| June | 116 | December | 106 |

From these figures it would appear that the maximum mortality was in spring, the minimum in summer.

The accompanying figures show the proportion, *per cent.*, of *recoveries* within different periods of time:—

| | Males. | Females. | Total. |
|---|---|---|---|
| Less than three months | 15·1 | 13·4 | 28·2 |
| More than three months and less than six months | 11·9 | 12·8 | 22·7 |
| More than six months and less than one year | 11·5 | 11·6 | 23·5 |
| More than one year and less than two years | 6·2 | 5·3 | 11·6 |
| More than two years and less than three | 2·4 | 2·4 | 5·3 |
| More than three years and less than four | 1·3 | 0·9 | 27·9 |
| More than four years and less than five | | | 12·3 |
| More than five years and less than six | | | 9 0 |

| | |
|---|---|
| More than six years and less than seven | 4·9 |
| More than seven years and less than eight | 3·2 |
| More than eight years and less than nine | 2·4 |
| More than nine years and less than ten | 4·5 |
| More than ten years | 0·8 |

Thus, more than one-fourth of the recoveries took place within three months after admission into an asylum; one-half within the six months; and three-fourths within twelve months.

Of the admissions during the five years, 22·0 per cent. (11·9 males, 10·3 females) had been in an asylum once before; 2·5 per cent. (1·1 males, 1·4 females) had been twice before; and 1·1 per cent. (0·9 males, 0·8 females) had been thrice.

The form of mental disease and special tendencies, as well as the probable curability and incurability of the patients in the different asylums on the 1st January, 1857, is shown in the subsequent summary.

The figures show the percentage upon the total number of patients, namely, 3824, (1949 males and 1875 females.)

| | Males. | Females. | Total. |
|---|---|---|---|
| Mania, Acute | 13·4 | 13·1 | 13·2 |
| " Ordinary | 14·7 | 13·9 | 14·3 |
| " Chronic | 31·1 | 26·9 | 29·0 |
| Moral Insanity | 4·2 | 3·6 | 3·9 |
| Melancholia, (whether or not attended with delusions or occasional excitement) | 9·3 | 14·9 | 12.1 |
| Congenital idiocy | 3·1 | 1·4 | 2·3 |
| Congenital imbecility | 2·9 | 3·0 | 2·9 |
| Other forms of mental disease:— | | | |
| Dementia | 21·0 | 27·7 | 21·9 |
| Epileptics | 8·7 | 4·8 | 6·7 |
| Dangerous to others | 12·6 | 10·1 | 11·1 |
| Suicidal | 3·6 | 2·9 | 3·2 |
| Of dirty habits | 12·9 | 13·2 | 13·1 |
| Received from jails:— | | | |
| Dangerous lunatics | 23·7 | 14·2 | 19·0 |
| Criminal lunatics | 1·6 | 0·3 | 0·9 |

The probability of *recovery* among the whole of these cases was as follows:—

| | | | |
|---|---|---|---|
| Probably curable | 28·5 | 32·6 | 30·5 |
| Probably incurable | 71·4 | 67·3 | 69·4 |

The percentage of patients paid for by their friends was, 1·5—males, 2·0; females, 1·0.

The assigned *causes* of the mental disorder in the cases admitted into the district asylums during the five years 1852–56 may be divided into two classes, the *physical* and the *moral*. In the *first class* there may be enumerated, of those cases in which a cause is distinctly specified, 722 males and 707 females; in the *second class* may be summed up 262 males and 461 females. The principal physical causes mentioned, and the number of cases in which they are supposed to have been effective, are as follows:—

| | Males. | Females. |
|---|---|---|
| Heritage | 360 | 333 |
| Epilepsy | 81 | 44 |
| Intemperance and dissipation | 40 | 60 |
| Mechanical injuries | 63 | 17 |
| Injuries of the head | 28 | 4 |
| Fever | 31 | 52 |
| Child-birth | — | 58 |
| Cold, excessive work, and destitution | 18 | 10 |

The principal moral causes named are:—

| | | |
|---|---|---|
| Grief | 47 | 85 |
| Fright | 30 | 46 |
| Grief and fright | 30 | 46 |
| Total from these emotions | 107 | 177 |
| Loss of property | 60 | 40 |
| Jealousy and love | 30 | 45 |
| Disappointment | 19 | 41 |
| Death of a member of the family, or of friends | 10 | 44 |
| Death and emigration of a relative or friend | 4 | 32 |
| Domestic trials | 16 | 19 |

The *occupation* of the lunatic prior to his insanity is mentioned in 3794 instances, of which the accompanying list gives a brief summary:—

| | |
|---|---|
| Laborers | 1445 |
| Servants | 523 |
| Agricultural pursuits, farming, gardening, etc., (farmers, 372) | 507 |
| Handicraftsmen | 427 |
| Tradesmen | 239 |
| Dress and bonnet makers, and needlewomen | 168 |
| Policemen, soldiers, excisemen, and pensioners | 151 |
| Professions | 124 |
| Clerks | 72 |
| Shipwrights, sailors, boatmen, and fishermen | 44 |
| Paupers | 29 |
| Gentry | 19 |
| Engaged about horses | 18 |
| Prostitute | 1 |

The data are not given by which the relative proportion of these figures to the average number of individuals living in each of the specified classes of occupation could be ascertained for the period in question.

The value of the statistics furnished by the district asylums is in several respects much diminished from the want of a comprehensive system of registration common to all the asylums. But this is a defect which is far from being peculiar to the Irish asylums.

